# ‘Should I Stay, or Should I Go?’ Psychological Distress Predicts Career Change Ideation among Intensive Care Staff in Lithuania and the UK Amid COVID-19 Pandemic

**DOI:** 10.3390/ijerph18052660

**Published:** 2021-03-06

**Authors:** Ieva Norkiene, Lina Jovarauskaite, Monika Kvedaraite, Encarl Uppal, Mandeep Kaur Phull, Heidi Chander, Kathryn Halford, Evaldas Kazlauskas

**Affiliations:** 1 Clinic of Anaesthesiology and Intensive Care, Institute of Clinical Medicine, Faculty of Medicine, Vilnius University, M. K. Ciurlionio Str. 21, LT-03101 Vilnius, Lithuania; ievanork@gmail.com; 2 Center for Psychotraumatology, Institute of Psychology, Vilnius University, M. K. Ciurlionio Str. 29, LT-03100 Vilnius, Lithuania; kvedaraite.monika.8@gmail.com (M.K.); evaldas.kazlauskas@fsf.vu.lt (E.K.); 3 The Royal London Hospital, Barts Health NHS Trust, Whitechapel Road, Whitechapel, London E1 1FR, UK; encarl.uppal@nhs.net; 4 Barking Havering and Redbridge University NHS Trust, Rom Valley Way, Romford Essex, London RM7 0AG, UK; mandeep.phull@nhs.net (M.K.P.); heidi.chandler@nhs.net (H.C.); kathryn.halford@nhs.net (K.H.); 5 Queen Mary University of London, Mile End Rd, Bethnal Green, London E1 4NS, UK

**Keywords:** COVID-19, healthcare, mental health, burn-out

## Abstract

The COVID-19 pandemic had a significant effect on healthcare globally. Additional pressure created by coronavirus adversely affected the mental health and psychological well-being of healthcare workers, leading many to question their desire and willingness to continue working in healthcare. This study aimed to identify predictors for career change ideation among healthcare professionals in two countries; Lithuania and the United Kingdom amid the coronavirus pandemic. In total, 610 healthcare professionals from Lithuania and the UK (285 and 325, respectively) participated in a survey from May to August 2020. Psychological distress and psychological well-being were measured using the self-report scales “DASS-21” and “WHO-5”. Almost half of the sample (49.2%), 59.6% and 40.0% in Lithuanian and the UK, respectively, exhibited career change ideation, the country effect was significant (*AOR* = 2.21, *p* < 0.001). Stronger ideation to leave healthcare was predicted by higher levels of depression (*AOR* = 1.10, *p* = 0.005), stress (*AOR* = 1.10, *p* = 0.007), anxiety surrounding inadequate personal protective equipment (*AOR* = 2.27, *p* = 0.009), and lower psychological well-being scores (*AOR* = 1.10, *p* = 0.007). We conclude that psychosocial support must be provided for healthcare professionals to prevent burnout and loss of staff amid the pandemic.

## 1. Introduction

Officially declared a pandemic on 11 March 2020 [1], Coronavirus Disease 2019 (COVID-19) placed unprecedented levels of pressure on healthcare systems worldwide. As of March 2021, there have been over 113 million confirmed cases and 2.5 million deaths internationally [2].The increased demand for Intensive Therapy Unit (ITU) services forced the temporary restructuring of healthcare systems. Healthcare workers (HCW) encountered new challenges because of redeployment to unfamiliar specialties/areas to match this demand.

The first studies investigating the link between coronavirus and mental health in China reported that staff who treated patients with COVID-19 experienced high levels of anxiety, stress and depression [3,4,5]. Increased workload, social isolation, challenges in personal safety, and caring for confirmed cases were shown to have a significant impact on mental health morbidity and psychological well-being. Moreover, studies showed that compared to the general population or even non-healthcare related hospital staff, HCWs displayed significantly higher rates of various mental health problems during the pandemic [6,7]. Similarly, Italian HCWs who treated COVID-19 patients experienced high levels of psychological distress [8]. This was in part exacerbated by their own health anxieties, e.g., fear of contracting coronavirus or infecting family members and cohabitants [9]. A survey of 3000 American HCW demonstrated a high incidence of physical and psychological risk [10] in those exposed to coronavirus. An increased risk of anxiety, burnout and depressive symptoms was associated with fear of self-infection, self-isolation, and co-habitants moving out of the HCW’s house. A study conducted in Cyprus also showed that depressive and PTSD symptoms of HCWs increased even if the spread of the COVID-19 in the country at the time was relatively low [11].

HCWs experienced higher perceived levels of threat due to fear of contracting coronavirus in comparison to the general population; a factor that increased their likelihood of developing COVID-19 related psychological stressors (COVID-19 Psychological Research Consortium (C19PRC) [12]. However, studies showed that not only health-related factors are directly related to higher levels of stress in HCWs but also work-related factors, such as working with COVID-19 infected patients, increased workload or working in the departments directly related to treating COVID-19 infected patients (e.g., respiratory, ICU, infectious disease) [13]. Work-related factors during the COVID-19 pandemic has also caused an increase in resignations, especially from nurses, because of overwork [14].

The link between healthcare provision during a viral pandemic and increased mental health morbidity has been identified previously [15]. Open communication, access to personal protective equipment (PPE), adequate rest, and both practical and psychological support were found to be effective protective strategies in previous viral outbreaks. In addition to these strategies, during the COVID-19 pandemic, professionals also strongly suggest mandatory psychological support services and mandatory occupational health surveillance programme [16]. 

The aim of this study was to measure pandemic driven psychological distress and ideation to change careers in a diverse group of HCW from two countries: the UK and Lithuania. Given the difference in numbers of cases and therefore pandemic related workload, we expected the UK to experience higher levels of psychological distress and career change ideation. Using this data, we also aimed to determine what factors predicted ideation to change career in HCWs facing the COVID-19 pandemic in the studied sample. 

## 2. Method

### 2.1. Participants

A total of 610 HCWs participated in the study; 325 British and 285 Lithuanian. The mean age of the total sample was 40.40 (*SD* = 11.20). 238 (73.2%) of British and 242 (85.3%) of Lithuanian participants were female. The UK sample comprised of SHO/core trainees (17.8%), specialty trainees (8.0%), fellow/specialty doctors (4.3%), consultants (10.5%), nurses (52.3%), nurses in training (0.9%) and health care assistants (6.2%). The Lithuanian sample was comprised of medical doctors (60.0%), nurses (27.0%), specialty trainees (11.5%), health care assistants (1.1%) and medical students (0.4%). Candidates in both groups worked within a variety of specialties, i.e., ITU, Anesthesiology, Cardiology, Psychiatry, Internal Medicine, etc. 

### 2.2. Procedure 

A survey was distributed to Lithuanian HCW online over July–August 2020. The survey was disseminated across the country using social media to reach professionals groups of HCWs and internal emails of specialized medical associations. At the time of data collection, the Lithuanian stringency level was relatively low (25.93–28.70) [17], meaning there were recommendations to avoid large gatherings and stay at home if possible, but there were no restrictions for workplaces and public events up to 1000 people. There were reported 1818–2906 total Coronavirus cases in Lithuania during the data collection period, with an average of 45 new cases every day [18]. 

UK data was collected via the completion of an electronic or paper survey. The collection period took place over May–July 2020 within a large acute NHS trust comprising two hospitals, employing over 6500 staff members. The electronic survey was distributed via social media, the hospital trust website, and an internal email server. Paper surveys were completed in private and sealed in an envelope before being manually entered into the online survey platform by the study team. At the time of data collection, the UK stringency level was relatively high (64.35–79.63) [17], meaning all public events were cancelled, there were restrictions on gatherings of more than 10 people, schools and the majority of workplaces were required to close. There were reported 160,769–303,181 total Coronavirus cases in the UK during the data collection period, with an average of 3000 new cases every day in May, 1500 new cases every day in June and less than 1000 new cases every day in July [18]. 

In both countries, the study was conducted over a single recruitment period with two additional reminders sent to encourage participation. All participants of this study provided written informed consent before completing the survey electronically or on paper. The same secure online survey platform was used in both countries, which allowed IP filtering to prevent duplicate responses. All collected data was anonymized and the answers provided online were not linked with participants IP addresses. Approval for this study was granted by the Institutional Psychological Research Ethics Committee (Lithuania) and NHS Health Research Authority (United Kingdom).

### 2.3. Measures

#### 2.3.1. Profession-Related Factors 

HCW career change ideation was assessed by one question with a binary response option: ‘Have you thought, at any point in the last month, about changing your career and working outside healthcare?’ (No/Yes). Participants specialty (i.e., ITU, Cardiology, etc.) and time spent working in healthcare (years’ experience) was also collected.

#### 2.3.2. Concerns about COVID-19 

Four statements were designed by the study team to assess HCW anxieties surrounding coronavirus: (1) ‘I do not feel anxious, I trust the protective equipment’, (2) ‘I have a constant fear of being infected, but I believe the disease will be easy’, (3) ‘I have a constant fear of infection and death’, (4) ‘I worry my loved ones might be infected‘. All four statements had a binary response option: ‘Yes/No’, indicating their agreement or disagreement with the statement.

#### 2.3.3. Depression, Anxiety, and Stress

The Depression Anxiety Stress Scale (DASS-21) [19,20] was used to measure the mental health morbidity of the study population. The DASS-21 is comprised of three subscales; each containing 7 questions (21 questions in total) which specifically screen for symptoms of depression, anxiety, and stress. A 4-point Likert scale ranging from ‘Never’ (0) to ‘Almost always’ (3) was used to measure the participants’ response to each question. Each of the three components (depression/anxiety/stress) are given a score, calculated by summation of the responses to each subscale question. The severity of each component was graded by its score: depression (normal/mild <7; moderate 7–10; severe >11), anxiety (normal/mild <6; moderate 6–7; severe >8), stress (normal/mild <10; moderate 10–12; severe >13). Cronbach alpha internal consistency for depression, anxiety, and stress subscales were 0.90, 0.81, 0.89 in the UK sample and 0.91, 0.77, 0.87 in the Lithuanian sample.

#### 2.3.4. Well-Being 

Perceived well-being was assessed using the WHO-5 Well-Being Index (WHO-5) [21]. The WHO-5 comprises 5 questions with a 6-point Likert scale ranging from; ‘At no time’ (0) to ‘All of the time’ (5). Cronbach alpha internal consistency in the UK and Lithuanian was 0.92 and 0.89, respectively.

#### 2.3.5. Data Analysis

Data analysis encompassed two stages and was conducted with IBM SPSS© 25.0. In the first stage, a univariate statistical analysis was used to compare responses of UK HCWs to Lithuanian HCWs. This analysis focused on sociodemographic characteristics, profession-related factors, concerns surrounding COVID-19, and mental health morbidity. The second stage involved using binary logistic regression analysis to identify which factors were associated with the desire to change career. We performed a univariate binary logistic regression to estimate the odds ratios of the individual study variable on career change ideation. Further, we performed multivariable binary logistic regression by including all the predictors in the model simultaneously [22] to estimate adjusted odds ratios. All the study variables were included in the binary regression model in the next step to deal with the potential overfitting [23]. Moreover, multivariable binary logistic regression, compared to univariate, enabled us to evaluate the effect of the variable with regard to the interaction of other predictors in the model. 

## 3. Results

### 3.1. Sociodemographic Characteristics of the Sample 

The sample characteristics are presented in Table 1. The average age of UK respondents was 40.50 yrs (*SD* = 11.60) with a range of 22–68 yrs, and Lithuanian respondents of 40.30 yrs (*SD* = 10.80) with a range of 20–70 yrs. There was no significant difference in average age between the two groups *t*(605.458) = 2.34, *p* = 0.815. In both groups, two-thirds of participants were in a long-term relationship (*χ*^2^(1) = 1.05, *p* = 0.337). A higher proportion of UK respondents were male *χ*^2^(1) = 12.36, *p* < 0.001.

### 3.2. Work Experience and Career Change Ideation

Almost half of the 610 participants had thought about changing career in the last month. Overall, 59.6% of Lithuanian HCW considered leaving healthcare vs. 40.0% of UK HCW (χ^2^(1) = 23.46***). Over 50% of all HCWs surveyed had >10 yrs work experience, and the average years worked in healthcare were the same in both groups (see Table 1).

### 3.3. Concerns Regarding the COVID-19 

Significantly more Lithuanian HCW (48.1%) than UK HCW (33.5%) trusted PPE (χ^2^(1) = 13.33, *p* < 0.001). UK HCW were more likely to experience “constant fear of infection and death” (18.8% vs. 5.6%) χ^2^(1) = 23.83, *p* < 0.001), and “worry their loved ones might be infected” (59.1% vs. 45.3%) χ^2^(1) = 11.62, *p* = 0.001). Significantly more Lithuanian participants (25.6%) experienced fear of “being infected but believed the overcoming the disease would be easy”, in comparison to 17.2% of UK participants (χ^2^(1) = 6.40, *p* = 0.013).

### 3.4. Mental Health of Medical Staff Members

The severity of depression in UK and Lithuania HCWs were distributed as follows: normal/mild—271 (83.4%) and 215 (75.4%), moderate—23 (7.1%) and 39 (13.7%), severe—31 (9.5%) and 31 (10.9%), respectively. Majority of all participants report normal/mild levels of anxiety (285 (87.7%) in the UK; 258 (90.5%) in Lithuania), but few experienced severe anxiety (12 (3.7%) in the UK, 7 (2.4%) in Lithuania). Normal/mild, moderate, and severe rates of stress were observed in the UK (241 (71.1%), 45 (13.9%), 39 (12.0%)) and Lithuanian HCW (148 (51.9%), 69 (24.2%), 58 (23.9%)).

A comparison analysis (see Table 1) showed that Lithuanian HCWs experienced higher rates of depression (*t*(608) = −3.45, *p* = 0.001) and stress (*t*(608) = −7.49, *p* < 0.001) compared to UK HCWs. However, there was no significant difference in anxiety levels between the two groups *t*(607.825) = −1.27, *p* = 0.204). Additionally, UK HCW reported higher psychological well-being scores than Lithuanian HCW *t*(607.657) = 2.79, *p* = 0.005.

### 3.5. Predictors of Career Change Ideation

Univariate binary logistic analysis revealed that lower age, being in a long-term relationship, working in Lithuanians vs. UK healthcare system, COVID-19 concerns (distrust in protective equipment, worries about infecting the closed ones), and all mental health indicators of the study significantly predicted career change ideation (see Table 2).

Multivariable binary logistic regression analysis (Nagelkerke *R*^2^ = 0.38) showed being of younger age (*AOR* = 0.97, *p* = 0.003), working in a Lithuanian healthcare system (*AOR* = 2.21, *p* < 0.001), having low trust in the effectiveness of PPE (*AOR* = 2.27, *p* = 0.009), scoring highly on DASS-21 depression scale (*AOR* = 1.10, *p* = 0.005), scoring highly on DASS-21 stress scale (*AOR* = 1.10, *p* = 0.007), and low WHO-5 psychological well-being scores (*AOR* = 0.97, *p* < 0.001) are all significantly associated with an increased desire to change career (see Table 2). Being in a long-term relationship had a marginal effect on the dependent variable (*AOR* = 1.58, *p* = 0.050). The Hosmer-Lemeshow test indicated a good model fit for overall binary logistic regression model, *χ2*(8) = 7.69, *p* = 0.485.

Gender, the strength of concerns regarding COVID-19 (i.e., fear of infection of self or family members) and scoring highly on the DASS-21 anxiety scale were not shown to have a significant impact on the desire to change career.

Univariate vs. multivariable logistic analysis yielded similar findings. However, we also found that after controlling for all the study variables, worries about the infection of the loved ones and anxiety was not a significant predictor for the career change ideation.

## 4. Discussion

This is one of the first studies to compare the psychological burden and mental health in HCW during the COVID-19 pandemic across two countries. We identified a high prevalence of moderate-severe depression, stress, and anxiety in the UK and Lithuanian HCWs. These findings are comparable to other studies which explored the psychological distress of HCW in the UK and other countries [24] during the ongoing COVID-19 pandemic. The study is also in line with studies of previous pandemics which reported a high prevalence of mental disorders among the pandemic affected HCW [24].

Our study revealed cross-country variations in psychological distress levels. UK data collection took place during May–July 2020. At this time there was a higher number of coronavirus positive patients circulating in the population in comparison to the July-August 2020 period that the Lithuanian data collection was completed [17]. One would expect the mental health disease burden to be greater in the UK data set, matching the increased pandemic related workload likely encountered by UK professionals participating in the study. In fact, Lithuanian HCW reported significantly higher levels of depression and anxiety, and lower psychological well-being scores in comparison to UK HCW. This finding indicates that factors other than external stressors and increased workload can influence psychological distress in HCWs. One explanation for this could be the lack of availability and usability of psychological support systems for HCWs [14]. We hypothesize that Lithuanian healthcare institutions may not yet have well-developed and functioning psychological staff support systems in operation, causing a higher level of psychological distress amongst its staff. Previous studies confirm this hypothesis and have demonstrated high levels of burnout among Anaesthesia and Intensive Medicine physicians [25].

We also found a large percentage of UK and Lithuanian HCWs exhibited career change ideation. Strong ideation to leave healthcare was associated with higher levels of depression, stress, anxiety surrounding inadequate personal protective equipment, and lower psychological well-being scores. Psychological distress predicted career change ideation, as well as anxiety surrounding inadequate protective equipment. Our results are consistent with a recent survey of almost 1000 healthcare professionals, which showed one in five UK healthcare workers were more to likely leave their role after the pandemic [26].

We hypothesize that ideation to leave healthcare is an indicator for professional burn-out, commonly associated with an increased workload, lack of support, and higher levels of stress. There are several studies proving that healthcare professionals are at high risk for burn-out [27,28]. However, no comparable studies are exploring the ideation to leave healthcare during the pandemic.

## 5. Limitations

One limitation of the study is the cross-sectional design, as there was no pre-test data available for career change ideation rates prior to the pandemic. This could be solved by using a longitudinal study design, which would allow us to measure how pandemic related stress affected rates of career change ideation during the pandemic. Results comparing HCWs in Lithuania and the UK should also be interpreted with caution due to the sampling procedure. The sampling procedure was different in the UK (restricted to a single region) and Lithuania (nationwide), and we also could not calculate the response rate due to our sampling procedures. Furthermore, we used a single-item as a career change ideation measure. The purpose of this was to reduce the length of the survey, as we feared a long questionnaire could deter professionals from participating. If we had the opportunity, we would consider using more detailed measures, including those focusing on burn-out to develop a better understanding of the concerns amongst HCWs working during the pandemic. Finally, while the study sample is large enough to run data analyses with sufficient statistical power, the sample size for both Lithuanian and UK groups is small. A larger study with a larger sample size including other countries would provide a better insight into the mental health of HCWs during the pandemic, and increase the accuracy of our inferences.

## 6. Conclusions

Our study has demonstrated that the mental health and psychological well-being of healthcare professionals have been adversely affected by the global pandemic. Around half of the study participants from the two countries with diverse histories and healthcare systems reported similar proportions of career change ideation. If these individuals were to act on their ideation, this could create significant problems in staff retention and healthcare provision in the UK and Lithuania. The threat of enormous staff shortages in such critical specialties as Intensive Care is concerning. Healthcare systems must take action to mitigate this threat, by providing new solutions for monitoring and managing psychological distress in its employees as well as adopting more psychological support for healthcare professionals (e.g., telemedicine, informal support groups) [29]. Psychological support for healthcare workers, among other things, implies a positive psychological climate in the workplace which encourages sharing the feelings or stressful experience, as well as facilitates seeking support among peers and supervisors, or psychological counselling if distress is high. Psychological support for HCW staff should not be limited to psychological counselling or well-being programs implementation but should include open and rapid communication to employees, development or improvement of mutual support skills in the team, referring to the open communication between employees, as well as their evolvement in the decision-making process, among the others.

## Figures and Tables

**Table 1:** Characteristics of the sample (*N* = 610).

				Study Location		
Variables			Total Sample (*N* = 610)	United Kingdom (*n* = 325)	Lithuania (*n* = 285)	Significance Statistics
Sociodemographic characteristics						
	Gender					
		Male	130 (21.3%)	87 (26.8%)	42 (14.7%)	*χ*^2^(1) = 12.36 ***
		Female	480 (78.7%)	238 (73.2%)	242 (85.3%)	
	Age					
	*M (SD)*		40.40 (11.20)	40.50 (11.60)	40.30 (10.80)	*t*(605.458) = 2.34
	Relationship status					
		In a long-term relationship	468 (76.7%)	244 (75.1%)	224 (78.6%)	*χ*^2^(1) = 1.05
		Not in a long-term relationship	142 (23.3%)	81 (24.9%)	61 (21.4%)	
Profession-related factors						
Work experience						
< 2 years			73 (12.0%)	40 (12.3%)	33 (11.6%)	*χ*^2^(2) = 1.75
2–10 years			204 (33.4%)	101 (31.1%)	103 (36.1%)	
> 10 years			333 (54.6%)	184 (56.6%)	149 (52.3%)	
	Had thoughts on changing career					
		No	310 (50.8%)	195 (60.0%)	115 (40.4%)	*χ*^2^(1) = 23.46 ***
		Yes	300 (49.2%)	130 (40.0%)	170 (59.6%)	
Concerns about the COVID-19						
	Trust in COVID-19 protective equipment					
		No	364 (59.7%)	216 (66.5%)	148 (51.9%)	*χ*^2^(1) = 13.33 ***
		Yes	246 (40.3%)	109 (33.5%)	137 (48.1%)	
	Fear of being infected, but having an easy disease					
		No	481 (78.9%)	269 (82.8%)	212 (74.4%)	*χ*^2^(1) = 6.40 *
		Yes	129 (21.1%)	56 (17.2%)	73 (25.6%)	
	Constant fear of infection and death from COVID-19					
		No	533 (87.4%)	264 (81.2%)	269 (94.4%)	*χ*^2^(1) = 23.83 ***
		Yes	77 (12.6%)	61 (18.8%)	16 (5.6%)	
	Worrying about loved ones being infected with coronavirus					
		No	289 (47.4%)	133 (40.9%)	156 (54.7%)	*χ*^2^(1) = 11.62 **
		Yes	321 (52.6%)	192 (59.1%)	129 (45.3%)	
Mental health, *M (SD)*						
	Depression		5.86 (4.58)	5.27 (4.49)	6.54 (4.59)	*t*(608) = −3.45 **
	Anxiety		4.53 (3.58)	4.36 (3.76)	4.73 (3.36)	*t*(607.825) = −1.27
	Stress		8.27 (4.47)	7.05 (4.49)	9.65 (4.03)	*t*(608) = −7.49 ***
	Well-being		45.32 (21.42)	47.56 (22.77)	42.78 (19.49)	*t*(607.657) = 2.79 **

* *p* < 0.05; ** *p* < 0.01; *** *p* < 0.001.

**Table 2:** Predictors of career change ideation and working outside the healthcare area in healthcare professionals (*N* = 610).

Variable	OR	95% CI	*p*	AOR	95% CI	*p*
Gender (male)	1.17	0.79–1.72	0.437	0.89	0.54–1.45	0.635
Age	0.87	0.96–0.99	<0.001	0.97	0.96–0.99	0.003
Being in a long-term relationship	1.44	0.98–2.10	<0.001	1.58	0.99–2.50	0.050
Country (Lithuania)	2.22	1.60–3.01	<0.001	2.21	1.44–3.39	<0.001
Distrust in the COVID-19 protective equipment	2.43	1.74–3.39	<0.001	2.27	1.23–4.20	0.009
Not believing the COVID-19 disease would be easy in case of being infected	0.71	0.48–1.05	0.090	1.39	0.82–1.22	0.221
No fear of infection and death from COVID-19	0.65	0.40–1.06	0.084	0.79	0.43–1.46	0.452
Do not worrying about loved ones being infected with coronavirus	0.49	0.35–0.67	<0.001	0.77	0.45–1.31	0.332
Depression	1.24	1.19–1.30	<0.001	1.10	1.03–1.17	0.005
Anxiety	1.19	1.13–1.26	<0.001	0.95	0.88–1.02	0.168
Stress	1.25	1.20–1.31	<0.001	1.10	1.03–1.18	0.007
Well-being	0.95	0.95–0.96	<0.001	0.97	0.96–0.99	<0.001

*OR* = odds ratio of univariate analysis; *AOR* = adjusted odds ratio; 95% CI = Confidence interval.

## Data Availability

The data presented in this study are available on request from the corresponding author. The data are not publicly available due to the data protection regulations.
